# Editorial: Epidemiology of Atypical Demyelinating Diseases

**DOI:** 10.3389/fneur.2021.662353

**Published:** 2021-05-03

**Authors:** Dalia Rotstein, Su-Hyun Kim, Yael Hacohen, Michael Levy

**Affiliations:** ^1^Department of Medicine, University of Toronto, Toronto, ON, Canada; ^2^St. Michael's Hospital, Toronto, Toronto, ON, Canada; ^3^Department of Neurology, National Cancer Center, Goyang-si, South Korea; ^4^Queen Square Multiple Sclerosis Centre, Institute of Neurology, University College London, London, United Kingdom; ^5^Department of Neurology, Massachusetts General Hospital, Boston, MA, United States

**Keywords:** NMO spectrum disorder, myelin oligodendrocyte glycoprotein antibody, EPI - epidemiology, demyelinating disease, optic neuritis, transverse myelitis

In Shakespeare a rose by any other name would smell as sweet, but in medicine names are chosen—and revised—to reflect the biologic underpinnings of the diseases they are affixed to. Over the last 15 years categorization of atypical demyelinating diseases has been transformed by the discovery of novel pathogenic antibodies. Identification of aquaporin-4 (AQP4) antibodies in patients with NMOSD has resulted in expansion of clinical phenotype beyond the optic nerve and spinal cord. In 2015, neuromyelitis optica was therefore renamed neuromyelitis optica spectrum disorder (NMOSD) (1). Myelin oligodendrocyte glycoprotein (MOG) antibody has since been observed in association with some seronegative cases of NMOSD, as well as with isolated and recurrent optic neuritis (ON), brainstem syndromes, encephalitis, transverse myelitis (TM), and acute disseminated encephalomyelitis (ADEM) (2). Diagnostic criteria for MOG antibody disease (MOGAD) have yet to be formally defined but current diagnostic schemata such as seronegative NMOSD are insensitive for clinical and radiographic features associated with positive serum MOG antibody testing (3). AQP4 and MOG IgG disease are now known to be marked by distinctive pathophysiology even where clinical phenotypes overlap: a primary astrocytopathy in AQP4+ disease vs. oligodendrocytopathy in MOGAD.

The new disease categorizations represent a seismic shift in the topography of the atypical demyelinating conditions and populations they affect—a shift with profound implications for hallmark demographic and clinical characteristics, prognostic factors, therapeutic algorithms, and approaches to repair.

This research topic focuses on the Epidemiology of Atypical Demyelinating Diseases as we embark on this new epoch. We would like to thank our authors and reviewers for their contributions, time, and insights.

## New Disease Definitions

Many pieces in this collection address how the new disease definitions have altered descriptive epidemiology of NMOSD, MOGAD, and other atypical demyelinating populations. Most notably, with incorporation of serum AQP4 testing in the 2015 diagnostic criteria, reported prevalence, and incidence of NMOSD have increased substantially in many world regions (Hor et al.). Racial differences in NMOSD prevalence have become more apparent with exclusion of MOG+ cases. Hor et al. report prevalence of NMOSD as ~1/100,000 in whites, ~3.5/100,000 in East Asians, and up to 10/100,000 in blacks. Data from a multi-national study (5) suggest that blacks were more likely to suffer from severe attacks [visual acuity ≤0.1 in at least one eye or Expanded Disability Status Scale (EDSS) score ≥6.0 at nadir] at onset with a further study from the US observing higher mortality rates in NMOSD patients of African ancestry (4). Further investigation will help guide resource distribution, intensity of therapy, and mitigation of racial inequities. Prevalence and incidence statistics for AQP4+ NMOSD and MOGAD will undergo additional revisions as existing antibody assays are improved, new autoantibodies are discovered, and regional registries are developed.

Testing for AQP4 and MOG antibodies has highlighted mimics previously mislabelled as NMOSD (Lechner et al.; Chhabda et al.). In double seronegative cases, localization and presumptive etiology may offer insights as we move toward targeted biomarker discovery and therapeutic approaches. Blackburn and Greenberg address the need for a new nomenclature for TM with distinction among infectious, para-infectious, idiopathic, and disease-associated inflammatory causes. Oliveira et al. discuss immune checkpoint inhibitors as the trigger for a range of demyelinating attacks, some due to unmasking of pre-existing CNS demyelinating, paraneoplastic, or other inflammatory conditions. Careful work-up including an accurate medical history, CSF studies, and serum antibody panels can help predict risk of recurrent disease.

## Prognostication

The need for evidence-based prognostic measures for NMOSD has risen to the forefront with our growing array of therapies, with AQP4 and MOG antibodies emerging as important predictors of disease course. In a systematic review and meta-analysis by Filippatou et al. relatively poor visual outcomes were noted in those with isolated ON with AQP4 in contrast to MOG autoantibodies. Risk of relapsing disease is likely lower in MOGAD, but may be higher in adult vs. pediatric patients and depend on duration of follow-up (6). The latter is illustrated in a survival analysis of a retrospective cohort of 21 MOGAD patients in Western Canada; probability of relapse was 0.43 at 1 year, but 0.63 at 4 years (Cross et al.). In another Canadian retrospective series of MOGAD, 7/9 experienced more than one attack, but EDSS scores were relatively favorable at last follow-up (1.0–3.0) (Alsharmrani et al.). The prognostic value of persistent MOG IgG positivity requires further investigation. Children with monophasic disease became negative for serum MOG IgG earlier than in relapsing disease (6), but seroconversion does not seem to wholly preclude the possibility of a subsequent attack (7).

Beyond autoantibodies, clinical, and demographic features may predict disease course and deserve further study. In AQP4+ NMOSD, there is a predilection for relapses to occur in the same location as the previous event (Muir et al.), although this may not be the case in MOGAD (Cross et al.). Age in NMOSD is predictive of attack location with ON being the most common localization prior to age 30 (8), and TM thereafter (Khalilidehkordi et al.). Age in MOGAD is likewise predictive of localization and phenotype with more ADEM attacks seen in children and focal ON or TM in adults (Parrotta and Kister) (6). Area postrema syndrome is extremely rare in MOGAD regardless of age, but a common presentation of AQP4+ NMOSD (Hyun et al.). Females may be at higher risk of relapse in AQP4+ disease (9). Chronic symptoms like pain and fatigue limit quality of life in NMOSD and MOGAD independent of attack frequency; we need to better understand who is at risk and how to treat these common complications (Asseyer et al.). MRI features including cervical cord atrophy (10) and optic nerve (11) and spinal cord lesion length (10) may predict disability outcomes but require validation. Serum biomarkers including glial fibrillary acidic protein (GFAP) and neurofilament light chain (NfL) are under investigation and may help guide therapy in the future; early data suggest that NfL and GFAP rise with relapses in AQP4+ disease, and NfL correlates with EDSS in MOGAD and AQP4+ NMOSD (12).

## Therapeutic Strategies and Potential for Repair

Three therapies with phase III randomized controlled trial evidence for treatment of AQP4+ NMOSD—eculizumab, inebilizumab, and satralizumab—were introduced in 2019–2020. These therapies prolong time to relapse and will likely improve patient outcomes although long-term efficacy and safety are unknown. Expansion in treatment options allows for new opportunities for personalized medicine in NMOSD. Age and gender (e.g., family planning) should be considered in therapy selection. D'Souza et al. review therapies with some evidence for safe use during pregnancy, including corticosteroids, azathioprine, eculizumab, rituximab, and tocilizumab. Future research will help clarify how personal characteristics, such as age, gender, and race, and clinical characteristics predict response and adverse event profiles for each therapy. Lifetime use of certain therapies may not be sustainable given higher risk of opportunistic infection in older individuals and regional resource availability and coverage climate. Development of treatment algorithms in NMOSD should also include evidence-based approaches to treatment de-escalation.

Appropriate therapy for MOGAD and seronegative NMOSD remains under investigation and represents a large unmet need. Recent evidence suggests efficacy of corticosteroids, azathioprine, and mycophenolate in MOGAD; benefits of rituximab have been less clear (13, 14).

The presence of specific target antigens in AQP4+ NMOSD and MOGAD may allow for novel therapeutic strategies through induction or restoration of immune tolerance—and obviate the need for long-term immunosuppression. Such strategies could include oral tolerization approaches as have been used to treat allergies, or a messenger RNA (or other) vaccine as has been explored in experimental autoimmune encephalomyelitis (15). These approaches have not yet proven successful in human autoimmune diseases but deserve further study. However, individual factors such as age, disease stage, and clinical phenotype could prove essential to the potential for repair (16).

What's in a name? For NMOSD and MOGAD, the new categorizations depend on pathogenic autoantibodies, but also have ushered in a new epidemiology with unique clinical characteristics, prognostic factors, and therapeutic strategies associated with each condition. Further epidemiologic research into these diseases will help refine management and improve outcomes. There remain many seronegative idiopathic demyelinating cases where an epidemiologic approach, with groupings by clinical phenotype, diagnostic test results, demographic features, relapse frequency, and any identified triggers, may focus efforts to identify additional antibodies and other underlying pathogenic mechanisms. For while insights into pathophysiology have led recent advances in treatment, epidemiology may provide the critical bridge to an enhanced, precision approach to the atypical demyelinating diseases.

## Author Contributions

All authors listed have made a substantial, direct and intellectual contribution to the work, and approved it for publication.

## Conflict of Interest

DR has received research support from the MS Society of Canada, CMSC, and Roche Canada. She has served as a consultant or speaker for Alexion, Biogen, EMD Serono, Novartis, Roche, and Sanofi Aventis. Related to this issue on NMO and MOG, ML received consulting fees from Viela Bio, Genentech, and Alexion. He also consulted for Sanofi, Quest Diagnostics, and UCB Pharamaceuticals. The remaining authors declare that the research was conducted in the absence of any commercial or financial relationships that could be construed as a potential conflict of interest.
